# Supplementary material for: Experiences of transgender persons in accessing routine healthcare services in India: Findings from a participatory qualitative study
Source: PLOS Glob Public Health. 2024 Feb 29;4(2):e0002933. doi: 10.1371/journal.pgph.0002933 (PMC10903866; doi:10.1371/journal.pgph.0002933)
Supplement: S1 File — (DOCX) [file pgph.0002933.s001.docx]

Guide for in-depth interview

इंटरव्यू के लिए गाइड

1. Access to healthcare

स्वास्थ्य सेवा का लाभ उठाने की सक्षमता

- 1. What forms of healthcare do you access? How regularly? Where do you access these from?

आप किन स्वास्थ्य सेवाओं का लाभ उठाते/उठा पाते है? कितने नियमित रूप से? आप इन सेवाओं को किस तरह से प्राप्त करते है?

- 1. What are the kind of thoughts that you would have in your mind before you visit a clinic or a hospital setting?

क्लिनिक या अस्पताल में जाने से पहले आपको क्या विचार/ख्याल आते हैं?

- 1. Can you describe your experience of visiting a clinic or a hospital setting from the time you enter till you exit?

क्या आप क्लिनिक या अस्पताल जाने का अपना पूरा अनुभव बता सकते है?

- 1. What is the attitude of the medical professionals and health facility staff towards you and how do they treat you?

अस्पताल के स्टाफ और स्वास्थ्य सुविधा कर्मचारियों का आपके प्रति कैसा व्यवहार हैं?

(अस्पताल में आपके साथ कैसा व्यवहार किया जाता है?)

- 1. How do the staff and other patients treat you while visiting a clinic or a hospital?

क्लिनिक या अस्पताल का दौरा करते समय वहा के कर्मचारी और अन्य मरीज आपके साथ कैसा व्यवहार करते हैं?

अस्पताल का स्टाफ और अन्य मरीज़ आपके साथ कैसा बर्ताव/व्यवहार करते है?

- 1. Are there reasons that prevent you from visiting a clinic or a hospital even when you need to?

क्या ऐसी दिक्कते हैं जो आपको ज़रूरत पड़ने पर भी क्लिनिक या अस्पताल जाने से रोकते हैं? ऐसी कौनसी चीज़ें/कठिनाइयां है जो आपको अस्पताल जाने से रोकती है या अस्पताल जाने में हिचक पैदा करती है?

1. Identities/पहचान/पृष्ठभूमि
   1. How do you identify yourself besides your gender identity?

अपनी लैंगिक पहचान के अलावा आप अपनी पहचान कैसे करते हैं?

- 1. Does coming from a rural/urban, poor/middle class, caste, religion background, sexual orientation, play a role in your access to treatment? How does this overlay with your identity as a transperson?

क्या ग्रामीण / शहरी, गरीब / मध्यम वर्ग से आना, जाति, धर्म /यौन रुझान आपके स्वास्थ सुविधाओं को प्राप्त करने में कोई भूमिका निभाती है? यह ट्रांस व्यक्ति के रूप में आपकी पहचान पर क्या प्रभाव डालता है?

1. COVID-19/कोरोना वायरस
   1. How did your experience accessing and navigating health facilities change during COVID19/lockdown/curfews?

कोरोनावाइरस / लॉकडाउन के दौरान स्वास्थ्य सुविधाओं तक पहुँचनेमें आपके अनुभव में क्या बदलाव आए?

- 1. Did you access specific services such as testing, treatment, vaccination during COVID-19? Kindly provide details.

क्या आपने कोरोनावायरस के दौरान परीक्षण (covid की जांच) और इलाज करवाई? क्या आप उसके बारे में बता सकते है?

- 1. Were you reached out to as part of any public health activity- surveillance, door- to- door surveys, door to door vaccination etc?

क्या आप तक कोई भी सार्वजनिक स्वास्थ्य गतिविधि, घर-घर सर्वेक्षण पहुंचा? (या आपका नाम किसी भी सर्वे में नामांकित था)?

1. Mental health/मानसिक स्वास्थ्य
   1. Have you ever accessed mental healthcare services? If yes, how was your experience. If no, are there reasons that prevent you from visiting a mental health professional even when you need to?

क्या आपने कभी मानसिक स्वास्थ्य सेवाएं ली है? यदि हाँ, तो आपका अनुभव कैसा रहा? यदि नहीं, तो आपने यह सेवाएं क्यों नही ली?

How do

you feel before/after visiting a clinic or a hospital? Does it make you sad/upset/anxious?

आप क्लिनिक या अस्पताल जाने से पहले / ाद में कैसा महसूस करते हैं? क्या यह आपको दुखी / परेशान / चिंतित करता है?

Guide for focus-group discussion

समूह चर्चा के लिए गाइड

1. Access to healthcare/स्वास्थ्य सेवा का लाभ उठाने की क्षमता
   1. What is the general experience of a transgender person visiting a clinic or a hospital?

एक ट्रांसजेंडर व्यक्ति का क्लिनिक या अस्पताल जाने का अनुभव अक्सर कैसा रहता है?

- 1. Why do you think it is challenging/difficult?

आपको क्या चुनौतीपूर्ण / कठिन लगता है?

- 1. Do you think transgender persons are able to access healthcare like other people? If yes/no, why/why not?

क्या आपको लगता है कि ट्रांसजेंडर व्यक्ति अन्य लोगों की तरह स्वास्थ्य सेवा का उपयोग करने में सक्षम हैं? यदि हाँ / नहीं, क्यों / क्यों नहीं?

- 1. How can this be improved? (eg: training doctors/nurses, separate ward, separate clinic or a hospital)

इसे कैसे सुधारा जा सकता है? (डॉक्टरों/नर्स की ट्रेनिंग, ट्रांसजेंडर व्यक्तियों के लिए विशेष वार्ड, क्लिनिक या अस्पताल)

1. Identity/पहचान/पृष्ठभूमि
   1. Other than being a transgender person do you think there are other reasons that determine the experience of a transgender person in a clinic or a hospital? If yes, what are they? How do they determine? (eg: region, religion, caste, skin colour, education, language, etc)

ट्रांसजेंडर व्यक्ति होने के अलावा क्या आपको लगता है कि ऐसे अन्य कारण हैं जो क्लिनिक या अस्पताल में ट्रांसजेंडर व्यक्तियों के अनुभव को /प्रभावित करते हैं? यदि हां, तो वो कौनसे कारण हैं? वे कैसे अस्पताल में ट्रांसजेंडर व्यक्तियों के अनुभव को निर्धारित करते हैं? (उदाहरण: क्षेत्र, धर्म, जाति, त्वचा का रंग, शिक्षा, भाषा, आदि)

1. COVID-19/कोरोना वायरस
   1. How has COVID-19 changed the experience of transgender persons in health facilities?

कोरोना वायरस ने स्वास्थ्य सुविधाओं में ट्रांसजेंडर व्यक्तियों के अनुभव को कैसे बदला है?

- 1. How was the experience of transgender communities in trying to access preventive, promotive and curative care during COVID-19 (for COVID-19 and other conditions)?

कोरोना वायरस महामारी के दौरान स्वास्थ्य सुविधाओं तक पहुचने की कोशिश में ट्रांसजेंडर समुदायों का अनुभव कैसा रहा?

1. Mental Health/मानसिक स्वास्थ्य
   1. Are transgender people able to access mental healthcare?

क्या ट्रांसजेंडर लोग मानसिक स्वास्थ्य सेवा का उपयोग करने में सक्षम हैं?

- 1. How does the experiences of transgender persons in health facilities affect them mentally/emotionally?

स्वास्थ्य सुविधाओं में ट्रांसजेंडर व्यक्तियों के अनुभव उन्हें मानसिक रूप से कैसे प्रभावित करते हैं?
