# Supplementary material for: Experiences of transgender persons in accessing routine healthcare services in India: Findings from a participatory qualitative study
Source: PLOS Glob Public Health. 2024 Feb 29;4(2):e0002933. doi: 10.1371/journal.pgph.0002933 (PMC10903866; doi:10.1371/journal.pgph.0002933)
Supplement: S1 Data — (DOCX) [file pgph.0002933.s002.docx]

**Supporting Data – Relevant Excerpts**

| **Excerpts** | **Participant ID** |
| --- | --- |
| If we don’t like the services received in a hospital, we go to another hospital. | A kinnar person in an FGD |
| If we want to make this structure more affirmative and more friendly in a safe environment, we have to have the medical curriculum changed. That's the start. So, I believe, that is the very, very first and very important thing that needs to be done | A kinnar person in an FGD |
| A lot of healthcare professionals don’t know better. Like us, cis people, have all grown up in a cis normative society, where cis is the “normal”, cisgenderism is normal, heterosexuality is the normal. So, people just don’t know better and as someone has already pointed out that we need more accessible, inclusive curriculum system for our future doctors and healthcare professionals. I have also experienced in a lot different aspect that the doctor doesn’t know better because they didn’t study those things when they were students. | A kinnar person in an FGD |
| I completely avoid government healthcare systems now. | A transman in an FGD |
| When people realize your identity is that of a transgender woman or that of a transgender man, there is a certain level of, there is a high level of inquisitiveness. Now, whenever there is inquisitiveness, it always borders on your right to privacy. | P1, a transwoman from Western India |
| If I was less passable as a woman and appeared more gender fluid or as non-conforming, perhaps the level of anguish I would have gone through would have been lesser. | P1, a transwoman from Western India |
| I told them that you know, I have an insurance card. And there was an argument between the nurse who was recording and [said] "aap logon ka bhi insurance hota hai, kya chutiyapanti hai?" So, words like *chutiyapanti*, *haraami* are the words that the nurses have used. | P12, transwoman from North India |
| There are few doctors who do not know how to behave with a trans [person]. They were quite rude and discriminatory. Just because you belong to a trans community, you were looked down. You are not respected and given the same respect others were getting. | P12, transwoman from North India |
| There have been instances where I have been sort of sexually given passes, like, either through the winks, or through the touches, which was inappropriate. I mean, I was not comfortable. And either through even talks, which I could find out that the person has sexual tendency, or favours that he's looking out from me. And those were something which I really don't entertain, and that really boils me.  I do not spare that person then. Then I go to any level, I don't care what that, person who that person is. I don't care. And I'm very glad about that. Unless I'm comfortable, I've never ever allowed anyone to have sexual favors from me. That's very clear. I mean, I don't know why, but it's there. And I'm not saying I'm Sati or, or, you know, untouched virgin women. No, I'm not. I was into sex work. And I am comfortable with sex work. So it's nothing like that also. But I'm very clear that when I say no, it means no. So, that's my stance, which I take very clearly. And that's when I lose my temper. I do get into fights, not physical fights, but my voice is enough to ensure that I get public attention and immediate support. | P12, transwoman from North India |
| If my community is misgendered, my community is the first person to lift their saris and to clap and abuse them rather than educating them. | P12, transwoman from North India |
| Any new healthcare settings that I approach, or any new sort of medical professional that I need, is quite hesitant, with the transgender identity especially, this is what I think. I don't know the reality, but my voice is quite masculine. So, I have a very heavy voice. And when I speak to people, the people frown, when I meet them for the first time, especially medical professionals ki ye bala hai kya. That's the only episode where I sort of used my tactics. But when I come to professional terms with them, and sort of very liberal, I do not use all those stuff. And it's, it's what, how I sort of led the awareness, being a trans identified individual. | P12, transwoman from North India |
| [As a transwoman] I used to get very irritated during my starting of my career when people used to call me sir. Even in the medical area, they see me wearing a saree, you can see my long hair and that I have done my surgery. And they of course, don't know whether I've had my surgery or not. But my appearance to them seems as if I am a feminine individual. But just because of my heavy voice, they would still address me as sir, mister and that that was my frustration point where I used to have arguments. Fight, not physical fight. But arguments with them. But over a period of time, I have become *samajhdaar* rather than them becoming *samajhdaar*, I have become more samajhdaar and I have sort of, you know, inculcated in myself that I need to be sensitive to explain them that it's not okay to address me with sir and mister. And those are my tactics nowadays. | P12, transwoman from North India |
| If we talk about changes, I think the healthcare system is also quite embedded with the entire structure of the society - also the system that exists on a larger, wider scale. | P12, transwoman from North India |
| I did share my sexual identity to the clinical psychologist who I was seeing in the government hospital. But the clinical psychologist again was a woman who had an individual space and there was much more privacy. The person was younger and I was hoping that the person would understand better than the old doctors who are sitting there - the psychiatrist. | P15, A non-binary trans person from South India. |
| Every time I tell somebody I am a queer trans, it is a huge amount of explaining and revisiting a lot things which I had to go through - which I don’t want to do with a doctor all the time. And for most of the kind of healthcare I have accessed it doesn’t matter if I am accessing as a queer trans or passing as a cis het man. | P15, A non-binary trans person from South India. |
| I can speak English and I am educated... [this] sort of makes it easier for me to navigate the power relationship between the doctor and patient | P15, A non-binary trans person from South India. |
| Every time I say that I am a queer trans person it leads to a lot more asking about my gender identity. Eventually everything becomes about my gender identity and sexual identity. | P15, A non-binary trans person from South India. |
| When the [Nurse or Doctor] used to see that our identity proof is on a male name… they used to giggle among themselves. There used to be a lot of misgendering | P16, A transmasculine person from South India |
| [My mother] would use the pronoun “they” in front of the doctor. That really took some burden off my shoulders. | P16, A transmasculine person from South India |
| I also informed [the gynecologist] that I am going to be transitioning and taking the injections. She laughed and made a joke about it and said, “Oh, so the next time you come here, you won't be allowed, because then you will look like a man.” That just made me feel uncomfortable and unwelcome there. | P16, A transmasculine person from South India |
| And many times I've avoided going to the emergency room just because I dont want to face all this misgendering and discomfort. So, I had a ruptured cyst in my ovaries. And I probably should have gone to the emergency room to check that out. But I was like, “No, I don't I'll just manage it at home.” | P16, A transmasculine person from South India |
| I'm very anxious and dysphoric before I enter the healthcare setting and while I'm receiving the healthcare. After going and getting whatever that I need, I feel a little hopeless. And I start questioning myself, whether I'm being dramatic. Whether I'm expecting too much from people. I feel down and sad after going to any healthcare setting. | P16, A transmasculine person from South India |
| And endocrinology, I met the doctor, I would say about three times before I decided to stop going to her - because she was being very transphobic in the sense that she had very binary idea of what a trans person should be. And she considered me a trans man when I'm, in fact, non binary trans person. And she was pushing for surgeries that I didn't want. So I just felt super uncomfortable and unsafe around her. | P16, A transmasculine person from South India |
| I used to go there to get my testosterone shots. Along with the note from my mental health professional saying that, you know, I'm a trans person, and I'm accessing these services. They would still misgender me and call me “she and Ma'am”. That just made me feel very uncomfortable and dysphoric. And so I was like, I'm just not going to go and I'll just do it myself. | P16, A transmasculine person from South India |
| Well, I would say, mostly the reason I went to the hospital was to get my testosterone shots administered--the injection administered once every month. However, I've stopped going to hospitals now and I'm doing it myself at home. There are two reasons. So, one reason that I stopped going to the hospital is obviously because of the pandemic. And the second reason is, I didn't feel comfortable there, because I was constantly being misgendered. | P16, A transmasculine person from South India |
| I mean, I'm very anxious and dysphoric definitely, before I enter the healthcare setting, and that is while I'm receiving the healthcare. After going and getting whatever I need I just feel a little hopeless. And I start questioning myself, whether I'm being dramatic. Whether I'm expecting too much from people. Yeah, I just feel down and sad after going to any healthcare setting. | P16, A transmasculine person from South India |
| She wanted me to do an ultra [sound] scan, breast scan and stuff just to rule out any abnormalities. So I went for the scanning. And they gave us only two options for gender to list on the form. So I put it as male. And I entered the scan room, and the doctor who was scanning was visibly confused. Because you know, I'm male and I have breasts, how is that possible? So he didn't say anything during the thing, but on the scan, it came out-- the report that came out. They diagnosed me with gynecomastia. So that kind of talks a lot about how they are not educated on the possibility of transgender people existing and the varying kind of bodies that we have. | P16, A transmasculine person from South India |
| ...at the reception, in the form, I gave my name as [name of the participant] because that is a name I associate with. And the receptionist asked me who is this for? I said this for myself”. But [they said] "you look like a male". I said, “so? there's a, you know, Identity Disorder". It is my way of representing myself. I was asked for an ID proof. I said like, “why do you need ID proof to provide a medical treatment?” They said "no". I thought that they cannot provide me any treatment. And after like arguing for 10-15 minutes the receptionist was like, I'll check with doctor and the doctor plainly denied seeing me. | P18, a transwoman |
| … there have been multiple instances where people normally are not asked for any ID proof [but trans persons are]. | P18, a transwoman |
| In one severe condition I was asked to consult a specialist, which was Rs. 3000 for consultation and after paying the fee the person said “okay, today, I'm busy, I can't see you. So please come on another time”. Within a couple of minutes, I was shooed away. | P18, a transwoman |
| People cannot understand pronouns. So even if I give a [name of the participant], first thing they will look at you very strangely. They will give, you know, strange expressions, they will make you feel awkward. And then they'll ask for ID proof. I mean, not everyone has a liberty of getting an ID proof updated with the right information and in recent, things have become more difficult. So, there have been multiple instances where people normally are not asked for any ID proof. I give my name as [name of the participant], people have asked me ID proof and there have been instances where I've been even denied treatment | P18, a transwoman |
| There have been instances when I was physically abused, as well. There have been instances of people staring, passing weird comments, saying words [that are] derogatory, calling out names loudly, using physical [advances], or trying to take advantage. So, is it really like I’m an untouchable human being? | P18, a transwoman |
| When I pass as cis-gendered, everyone is just like – ‘don't really care’. Everything goes on like for any other person... If it's an urgency, then I pass off as a cis identity. But if it is not a life-threatening issue, I usually present myself as a trans identity. | P18, a transwoman |
| They are clueless and so we go as a group. | P2, a transwoman from South India |
| So, we had to file a case against them. | P2, a transwoman from South India |
| I'll file an RTI somehow… And with two, three… we build pressure. | P2, A transwoman from South India who is an activist |
| Yeah, that's because they don't speak in English, they're not educated, they don't have the class privilege that I have. I have a caste dis-privilege, but I have a class privilege that compensates for it. Now who asks for caste in the private Medicare? That is not a factor there. So most of the other transgender persons they are people who are taking arms begging in trains and on the roads in traffic signals and shops and so on or doing sex work and they are much more marginalized, disenfranchised and marginalized than me... | P2, A transwoman from South India who is an activist |
| I've been in [name of the hospital] which is the government hospital for mental health in [state of South India], I have gone there for my gender dysphoria certificate. It was very frustrating because they were clueless, they sent me to the [name of a Research Institute] ... So we had to file a case against them, because we are asking them for gender dysphoria certificate. What does that have to do with intersex conditions? | P2, A transwoman from South India who is an activist |
| If my friend wasn’t there, I wouldn’t have been able to access all these mental healthcare, trans healthcare options. | A transmasculine person in an FGD |
| Community support is something I really believed in since the time I was in college. | P23, A gender fluid person from Northeast India |
| I always look for something which is more of a quick fix. So just visiting the pharmacy nearby my house and just discussing with the pharmacy people about, you know, what elements I have, and if they can give me some better medicines… | P23, A gender fluid person from Northeast India |
| So every time I'm unwell, I think it's not just the physical unwellness that I go through, but also the emotional and mental trauma of being neglected and ignored. | P23, A gender fluid person from Northeast India |
| The point is for them, their duty was not important for them... they said they will only come to my house once I paid 2500 rupees. I said "I don't have it right now I can arrange for it. But can one of you please come and take a look at it?" They said “no”. So I had to come back home. | P23, A gender fluid person from Northeast India |
| So empathy is one of the things they need to learn. And I feel only a few practitioners really understand that; not necessarily as a personal quality, but as a requirement of your own profession. | P23, A gender fluid person from Northeast India |
| Yes, we get permission. They [Nayak/guru] never refuse and if there is any problem, they come along with us to the hospital, also. | A kinnar person in an FGD |
| So, [a transgender person] had to do a lot of advocacies to try to meet multiple people, senior hospital staff, all the way to the health minister… after a lot of struggles one bed was allotted in a hospital for trans patients. | P36, a transmasculine person from South India |
| But when there is an occasion to be able to explain to them what being a trans person is and what they should be addressing me as that I make that effort because I think it's important to do that as well to bring about change. | P36, a transmasculine person from South India |
| The doctors were laughing at us. When I raised my voice, they started with the treatment. | P37, a kinnar person from Central India |
| Yes, I come across such people, who see us and realize who we are and after that keep annoying us by saying, “give us your number?”, “we want to meet you”, “when and where will you meet us?”, “where do you stay?” all this. | P4, a kinnar transwoman from Central India |
| If I go into the men's washroom, I can’t go with a saree. If I go into women's washroom, my voice gives it away | P40, a transwoman from North India |
| I quite like the way that [name of a renowned institution] has structured the inpatient wards … there is no gender segregation of the washrooms | P64, a transwoman from South India |
| Um, well, in terms of a hospital setting, first of all, the idea is that... they don't expect a trans person to turn up. I mean, they don't expect that a trans person can come to the clinic, to access health care and that they are a valid and, a very good person to treat or a person that they should treat. | P40, a transwoman from North India |
| I have never faced any sort of gender discrimination because my family has always supported me. There are many transgender people who don’t get family support or are thrown out of their homes or are treated very badly | P43, A transwoman from Central India |
| Other trans people said, “we go to him. And he's okay. He's fine. He's cool.” And that's how I heard his name… I always prefer visiting [health professionals] on the recommendation of fellow trans people. | P49, A transwoman from Eastern India |
| If there is some skin issues the first thing that comes to my mind is let's see if there is a home remedy. Let's see if somebody else has used some medication which I can use, which doesn't have side effects. Because you know, skin is something when you're going to a doctor, you have to - there is a lot of vulnerability. You may have to reveal some parts of your body. And there could be questions. So, I have to know that the doctor [who] is treating me is also not transphobic. And it's very difficult. How would we know? There is no badge that I'm not transphobic. So that something is difficult. So that's why home remedies are the first thing that comes to our mind. | P49, A transwoman from Eastern India |
| One incident makes you afraid and makes you apprehensive [that] many others may happen. And you try to protect yourself and make yourself safe in the best possible ways without the intervention of others. | P49, A transwoman from Eastern India |
| I choose the possibility of discrimination over irreversible harm. I have to choose the lesser evil, although mentally it's difficult. | P49, A transwoman from Eastern India |
| If they ask me to stop and get in line, all I do is say “I will clap loudly” and after that they don’t stop me. | P5, a kinnar person in an FGD |
| If I'm going to a gynaecological ward, I stand out. Because my partner is also a trans man both of us stand out. And then people just keep staring at us. So, my partner doesn't like going to hospitals, because bigger hospitals will have checkpoints, and those checkpoints are also segregated, and then the washrooms are an issue. | P50, a transmasculine person from North India |
| Then she wanted to examine me. And so, she was like “remove your pants”. There was no curtaining. Then I had to ask her for a towel... And, and then she came, and then she just put her finger inside. I was like, oh, my God, like, without even giving some warning. It just felt so violating | P50, a transmasculine person from North India |
| I don’t have to sit and explain to the doctor about what my gender is… So, every time I go to the doctor, it’s almost like I am doing a gender-sexuality session. And I don’t think that is something I want to deal with. | P50, a transmasculine person from North India |
| Going to a doctor is never my first instinct. I will go to a doctor when stuff gets worst… I try to self-medicate or do home remedies. | P53, A transman from Western India |
| So the anxiety is always there whenever I go through any kind of healthcare, or access any healthcare even when it came to mental health. | P53, A transman from Western India |
| In general, there is this look, it is not verbal, it exists in their body language. | P53, a transmasculine person from Western India |
| Most of the doctors do argue with us. But we can’t say anything to them. | P55, a kinnar person from Central India |
| Since health care services are a necessity… even though they are misgendering me and it makes me uncomfortable I still have to go there. | P57, A transmaculine from North India |
| When the trans person comes to the hospital setting, there is always a fleecing of money. Like, immediately, another 500 rupees will be added” | P40, a transwoman from North India |
| We normally don't experiment with new doctors, unless it is a very difficult situation. | P66, A transwoman from North India |
| Healthcare stigma is real. It is really happening. If you will walk with me into any public healthcare system, you see people staring at me. Forget about healthcare system, even if you walk on the road with me, people will stare, either identify you as a trans person, or they will contemplate why this you know, mad woman is working with a trans person. Because when we talk about transgender people, a lot of people have myths and misconception about them. Either they are afraid of transgender or they have a pity about transgenders that these people are there, or otherwise they have a sympathy towards [trans people]. Oh, these poor people. | P66, A transwoman from North India |
| The watchman never used to allow us to sit also. And they shoo away [trans] people like that. | P66, a transwoman from North India |
| The nursing staff are so insensitive. Insensitive at the highest level. They are very qualified, they're very educated, but sensitivity mein itne insenstitive hai that they have taken, they have the right to abuse on your face. Jo peeche gaali dete the, woh alag baat hoti hai, but they have the right to abuse you on your face, you know. And then I lose it, I completely lose, I don't tolerate anybody abusing me for no fault of mine. | P66, a transwoman from North India |
| The nurse will not even touch your body because they feel it is obnoxious to touch a body of a trans person. So, they used to call me for some patient saying that “please come and give her the sponge bath” and I usually say “why can't you give it? You are a nurse you should be giving”. Then they say “No, no, can you do it please? You will feel more comfortable doing it. People like us don’t feel so comfortable doing such things” | P66, a transwoman from North India |
| I was asked to undress in front of a male doctor. And it was very traumatizing experience for me. I kept asking for a female doctor. But they did not give me. | P66, a transwoman from North India |
| Because as a trans person, many times we try to avoid public health systems because of the judgments that we face over there, how people judge, how health care providers judge you, ask awkward questions right from the time when you enter into the health system, including the watchmen, the nurse and ward boys, how they treat you and all those kinds of things. And for us, even if you have to access like public healthcare system, generally, it is traumatizing because while you are waiting to meet to the doctor in the line and queue that's quite an embarrassing situation where people are looking at you, they are passing comments, they are making statements, they are making gestures, which are not appreciable to you. And it's always this feeling that somebody is talking behind you and you feel uncomfortable about it. That's the reason why most of the people including me, we go to the private medical health practitioners who are near to your area. | P66, a transwoman from North India |
| The other thing about it is that mainly majority of times that stigma discrimination within the healthcare system happens not by the senior doctors but that could happen by the class three class four healthcare providers. | P66, a transwoman from North India |
| I had taken a trans person who was burned because of an issue with her regular partner. We took her to the public health care facility. She was dying and the hospital was not able to take a decision on which ward she should be admitted - whether in a male or a female ward. | P66, a transwoman from North India |
| In the in-take form itself hospital should ask, which wards they are comfortable in. | Transmasculine person in an FGD |
| [Hospitals can have] segregated sections for every patient. [so that] every patient is guaranteed their privacy--whether it be using curtains or makeshift walls. | Transmasculine person in an FGD |
| [When asking to undress] the [male nurses] would shout saying “You are a boy, aren’t you? Then why are you shy?” So, that was very uncomfortable for me. So, I didn’t do it. But they kept pushing saying “uthao, uthao, uthao… [lift, lift, lift]”. And there was no woman around. I was not very comfortable with it, but I had to do it. | Transmasculine person in an FGD |
| ...My father is in [a prestigious job]. So, he had this respect that, “this is our officer” | Transmasculine person in an FGD. |
| When you try to tell them “Don’t use that name”, or “don't address me with those pronouns” instead of having the conversation about your illness, it turns into a moral policing conversation. | Transmasculine person in an FGD. |
| If we talk about changes, I think the healthcare system is also quite embedded with the entire structure of the society, also the system that exists on a larger, wider scale | Transmasculine person in an FGD. |
