# Supplementary material for: Experiences of transgender persons in accessing routine healthcare services in India: Findings from a participatory qualitative study
Source: PLOS Glob Public Health. 2024 Feb 29;4(2):e0002933. doi: 10.1371/journal.pgph.0002933 (PMC10903866; doi:10.1371/journal.pgph.0002933)
Supplement: Attachment — Submitted filename: Response to Reviewers.docx [file pgph.0002933.s003.docx]

**Response to Reviewers**

**Please see responses in red**

Thank you everyone for the encouraging feedback and specific comments. We have addressed all the comments and explained the same as per feedback received below.

**From editor:** It is a very interesting topic, however, some items could be improved (see reviewers comments). Some items should be more detailed and explained.
For instance, why the intersectional approach was chosen and what it is.
It is also recommended to explain how it is a participatory methodology. The only information is that you use interviews and focus groups. Is the manuscript part of a bigger project?
How impacted to data collection the merge of face-to-face interviews, zoom interviews, and telephone interviews?

**Response**: Thank you very much for your comments. We have responded and made appropriate changes including giving more details on the intersectional approach and the participatory methodology.

The manuscript is indeed part of a bigger project titled TransCare COVID19 which examined various aspects of transgender healthcare such as access to healthcare, mental health care and impact of COVID19. This paper focusses on the experiences in accessing general or routine healthcare services in India. The paper also explores the impact of these experiences and how communities navigate their healthcare access.

**Reviewer #1:** This is a well articulated article on a very timely issue. I suggest a minor revision on the following
1. Although published in other article from the same study, I suggest the authors to write more on intersectionality and community based participatory approach in the methods section, so that readers get adequate idea about these approaches from this article.

2. What is AS? please elaborate when first used
3. in page 10, line 200 in quote, give an explanation in parenthesis what was the 'they' in their language, I mean what exact word they used.
3. intersectionality is an important concept used in this paper. However, data and analysis on intersectionality in not adequate. I suggest to consider intersectionality in presenting and explaining each experiences in finding section 1, rather than making a small section of findings on 'intersectionality'.
4. this article is overloaded with quotes. However, overall the explanations of quotes should be more in-depth and detail. In these cases, the authors may like to remove some quotes and give more explanations and analysis of the quotes.
5. the authors can also draw a diagram/framework on challenges transpeople face in accessing heath care from an intersectional lens. This will help theorise the findings.

**Response:** Thank you very much for the appreciation and the comments. We have addressed all of them and explained as per serial numbers above.

1. We have written in little more detail about the intersectionality and community based participatory approach.
2. AS is the acronym for co-investigator Aqsa Shaikh. We have clarified that within the paper.
3. This interview was conducted in English itself and is transcribed verbatim. We have now added aspects of intersectionality in each section in the findings.
4. We have removed some quotes and added more explanation and analysis of the quotes.
5. We have tried to incorporate intersectionality as we understand it in the diagram

**Reviewer #2:** I would like to congratulate the authors on this study that fills an important gap in the literature. I strongly feel that the study makes significant contribution to the field by bringing alive the lived experiences of transgender community, and the direction it provides for the training of medical professionals in particular. I would like to make few minor but important points that I think would enhance the readability as swell as potential impact of the research work.

1. Authors should provide a brief narration with respect to the status the third gender and their rights in India and its potential implications. This background is important in light of the wide international readership of the journal.

2. Authors are requested to provide more details on the sampling. While authors mention that the participants are mostly urban, yet there seems to be heterogeneity in geographical and socio-economic background (including age groups) that would lead to varied experiences as in the last 5-6 years there has been few monumental developments in the country with respect to LGBTQIA+ community.

3. Authors make a very important point that multiple identities other than gender too shaped the experiences both positively and negatively. However, this seems inadequate based on evidence provided. Authors are requested to elaborate this section with more evidence.

**Response:** Thank you very much for the appreciation and the comments. We have addressed all of them and explained as per serial numbers above.

1. Thank you. We have added this.
2. Thank you for this comment. We have provided these details in the methods section. We did not collect information on the age of the participants and hence will not be able to present that in the paper.
3. We have added relevant evidence in different sections as per the earlier comment.

**Note**: In addition to responding to the comments from editors and revieiwers we have made some corrections in the manuscripts such as the table summarising the gender identities of the participants and the attribution of certain quotes were there were errors.
